# CT imaging biomarkers to predict severity and prognosis of pulmonary hypertension

**DOI:** 10.1371/journal.pone.0313235

**Published:** 2025-02-12

**Authors:** Do won Yoon, Yeonyee E. Yoon, In Chang Hwang, Wonjae Lee, Ki-Yeal Lee, Eun Ju Chun

**Affiliations:** 1 Department of Radiology, Seoul National University Bundang Hospital, Seongnam-si, Gyeonggi-do, South Korea; 2 Division of Cardiology, Department of Internal Medicine, Seoul National University Bundang Hospital, Seongnam-si, Gyeonggi-do, South Korea; 3 Department of Radiology, Korea University Kuro Hospital, Korea University College of Medicine, Seoul, South Korea; University of Dundee, UNITED KINGDOM OF GREAT BRITAIN AND NORTHERN IRELAND

## Abstract

**Purpose:**

To explore whether there are computed tomography (CT) imaging biomarkers that can stratify the severity of patients with pulmonary hypertension (PH).

**Methods:**

We retrospectively enrolled 144 consecutive patients with suspected PH who underwent CT pulmonary angiography and right heart catheterization (RHC). CT findings were analyzed by two observers for large vessel size [ascending aorta (A), pulmonary artery (P), inferior vena cava (IVC)], each chamber size, and septal angle. We investigated the associations between CT imaging parameters and the mean pulmonary artery pressure (mPAP) from RHC. During a median follow-up of 36 months, we observed major adverse cardiovascular events (MACE; all-cause mortality and hospitalization for PH worsening). Univariate and multivariate Cox regression models were used with hazard ratios (HR) and 95% confidence intervals (95% CI) to determine independent predictors of MACE in patients with PH.

**Results:**

Of 144 patients, 116 (80.2%) were diagnosed with PH based on an mPAP of 20 mmHg. Among CT parameters, P, P/A ratio, right ventricle (RV), and RV/left ventricle (LV) ratio were strongly correlated with mPAP values (Pearson’s correlation coefficient, all r < 0.001). During the follow-up period, 44 (30.6%) patients developed MACE (14 deaths and 30 hospitalizations). Using multivariate Cox regression analysis, the RV/LV ratio (HR 2.32; 95% CI: 1.17–4.59) was the best predictor of MACE, followed by age (HR 1.03, 95% CI;1.00–1.05) (all p < 0.05). Among various CT parameters, A, P, and P/A ratio showed excellent reliability with intraclass correlation coefficient ≥ 0.95.

**Conclusion:**

Among CT parameters, the RV/LV ratio was the most robust predictor of MACE in patients with PH, while the P and P/A ratios served as reliable indicators reflecting mPAP levels.

## Introduction

Pulmonary hypertension (PH) is a progressive and often fatal disease characterized by increased right ventricular afterload and right ventricular failure. It is a complex pathophysiological disorder caused by various causes, and its diagnosis requires a multifaceted, holistic, and comprehensive approach [1]. PH is diagnosed based on hemodynamic assessment by right heart catheterization (RHC) as a mean pulmonary artery pressure (mPAP) of more than 20 mmHg at rest [1]. Although RHC is the gold standard in the diagnosis of PH, it is invasive and cannot be used frequently or repeatedly [2]. Therefore, transthoracic echocardiography (TTE) has been recommended as the first-line, non-invasive diagnostic investigation in patients with suspected PH, because TTE provides comprehensive information on chamber morphology and function, and valvular abnormalities, and estimates of hemodynamic parameters [3]. However, in patients with obesity or advanced lung disease, echocardiographic evaluation is limited due to poor echo windows [4, 5].

Computed tomography (CT) is a well-known non-invasive imaging modality that provides several advantages in diagnosing PH, including the ability to rapidly visualize pulmonary vasculature and ventricular morphology and identify underlying causes, such as chronic thromboembolic pulmonary hypertension (CTEPH) [6]. Several studies using CT pulmonary angiography (CTPA) has reported that parameters such as higher pulmonary artery diameter, increased pulmonary to aorta ratio (P/A ratio), or dilated right ventricle (RV) can be radiographic signs of PH [7–12]. However, there is a paucity of data on CT parameters that reflect PH severity or predict major adverse cardiovascular events (MACE) in PH. Therefore, we aimed to identify if there are CT imaging biomarkers that can reflect the severity and prognosis of PH.

## Materials and methods

### Study population

**Fig 1:**
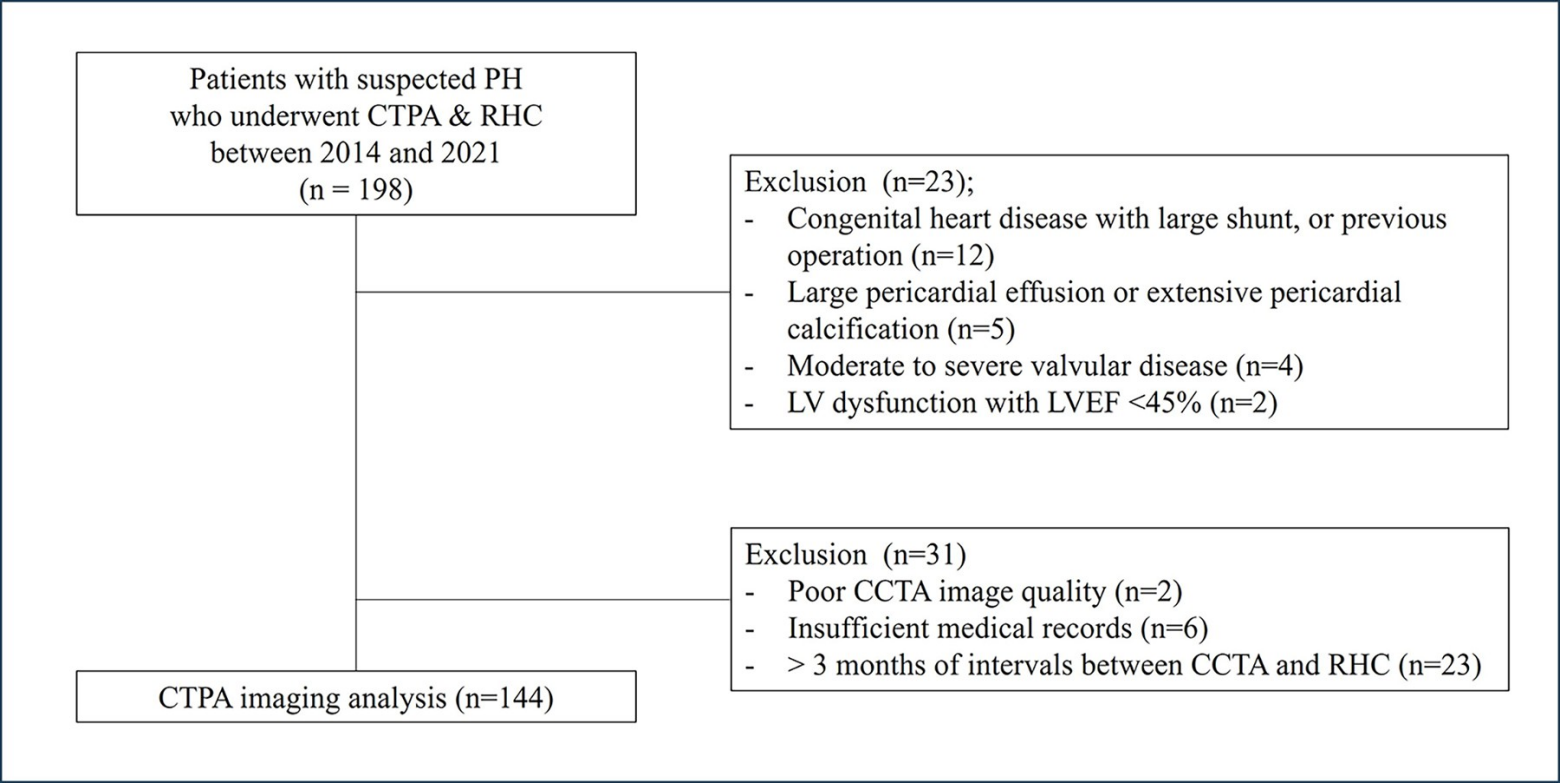
Flow chart of the study.

This retrospective study was approved by the local institutional review board, and informed consent was waived. We retrospectively analyzed data from consecutive patients who underwent both CTPA and invasive RHC for suspected PH between January 2014 to December 2021. Of these, we excluded patients with conditions that could alter the ventricular configuration or lead to errors in hemodynamic vascular assessment, including those who had undergone congenital heart disease with large shunt or cardiovascular surgery (n = 12), had a large pericardial effusion, or extensive pericardial calcification (n = 5), had moderate to severe valvular disease (n = 4), or left ventricular dysfunction with an ejection fraction of less than 45% (n = 2). We also excluded patients with poor CT image quality (n = 2), insufficient medical records (n = 6), and an interval of more than 3 months between CTPA and RHC (n = 23). Thus, a total of 144 patients were enrolled in the current study (Fig 1).

### Right heart catheterization

Venous access was obtained under local anesthesia. A balloon-tipped pulmonary artery catheter (6F, Arrow Single and Double-lumen Balloon Wedge-Pressure, Teleflex^®^) was used under fluoroscopic guidance, and Mac-Lab/SpecialsLab (software version 6.8, GE Healthcare) was used for monitoring, recording and analysis. The zero reference level was set at mid-chest level, RV pressure, PAP, pulmonary capillary wedge pressure, and right atrium (RA) pressure were measured at end-expiration. Blood samples were taken from chambers and vessels to estimate oxygen saturation. Fick’s method was used to determine cardiac output. Pulmonary vascular resistance was calculated as mPAP minus left atrium pressure divided by Q. PH was defined as > 20 mmHg.

### CT imaging protocol

CTPA was performed with a 256-slice multi-detector computed tomography (Brilliance iCT; Philips Healthcare, Cleveland, Ohio, USA) with 128 × 0.625 mm detector collimation and 270-ms rotation time. The whole chest was scanned from the lung apex to the diaphragm with a single breath-hold in a supine position at end-inspiration. After contrast agent (Iomeron 350, Bracco, Milan Italy) injection of a total volume of 3.5 mL/body weight (kg), administered at a rate of 3.5 mL/sec, scanning was automatically initiated 1.5 seconds after the contrast enhancement of the main pulmonary artery reached the preferred point (100 Hounsfield units [HU]). All CT images were reconstructed with a 1 mm slice thickness on the axial scan and a 3 mm thickness on the coronal scan. Additionally, maximal intensity projection technique with a slab thickness of 10 mm was used for pulmonary artery reconstruction.

### CT imaging analysis

**Fig 2:**
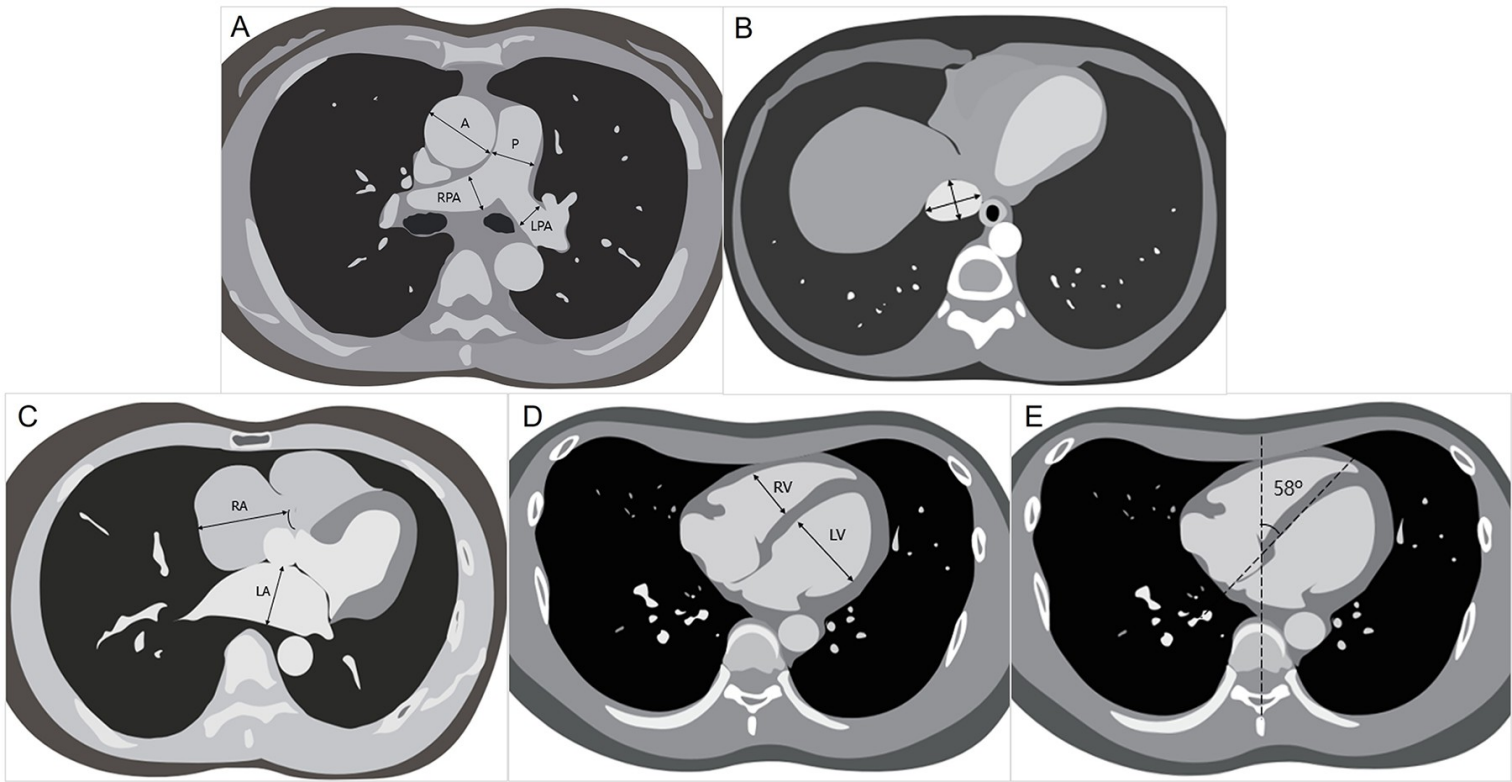
CT imaging parameters. A. The diameter of ascending aorta (A), main pulmonary artery (P), right pulmonary artery (RPA) and left pulmonary artery (LPA). B. Short and long diameter of inferior vena cava. C. Right ventricle (RV) and left ventricle (LV) diameter. D. Right atrium (RA) and left atrium (LA) diameter. E. Septal angle.

CT images were analyzed for large vessel dimensions and cardiac chamber size by two experienced cardiovascular radiologists (E.J.C. 15 years; D.W.Y. 3 years) using a workstation (IntelliSpace Portal version 11.0, Philips healthcare). Each CT image parameter was taken as the average value measured by the two radiologists (Fig 2).

### Large vessel measurements

The diameter of the main pulmonary artery (P) was measured perpendicularly to the vessel axis at its widest point on the axial image. Simultaneously, the diameter of the ascending aorta (A) was recorded at the same level as the main pulmonary artery measurement, allowing for the calculation of the P/A ratio. Additionally, the diameters of the right pulmonary artery (RPA) and left pulmonary artery (LPA) were measured at their widest points. Lastly, the long diameter of the inferior vena cava (IVC) was measured at its widest points, and the short diameter was also measured perpendicular to the long diameter in the same slice, because veins are observed as oval shapes due to less wall tension compared to arteries, which appear round.

### Cardiac chamber size and morphology evaluation

Cardiac chamber sizes and the septal angle were measured to estimate RV pressure and signs of RV overload. The maximum diameters of the RV and left ventricle (LV) cavities were measured in the axial plane at their widest points between the inner surfaces of the free wall and the interventricular septum. To assess the RA size on CT, the RA diameter was measured from the center of the tricuspid annulus to the lateral right atrial margin. The LA diameter was measured at the widest point antero-posteriorly on the axial image where the four pulmonary veins converge. The septal angle was defined as the angle between the interventricular septum and chest midline (the line between the spinous process of the vertebra and the midline of the sternum) on the axial image [13].

### Follow-up and outcomes

During a median follow-up of 36±27 months (range; 1–115 months), clinical data were acquired through the review of medical records or telephone contact with enrolled individuals by trained personnel. MACE included all-cause mortality and hospitalization for PH worsening [14].

### Statistical analysis

Continuous parameters are presented as mean ± SD, and nominal or ordinal parameters are given as n (%). For the comparison between CT imaging parameters and mPAP by RHC, the Pearson correlation coefficient (r) was used. Among clinical and CT parameters, univariate and multivariate Cox regression models were used with hazard ratios (HR) and 95% confidence intervals (95% CI) to determine independent predictors of MACE in patients with PH. Variables associated with survival with a P value <0.05 in the univariate analysis were included in the multivariate regression.

The Youden index derived from a receiver operating characteristic (ROC) curve analysis was used to determine the optimal cut-off values for predicting MACE. The inter-observer agreement of various CT imaging parameters was calculated using the intraclass correlation coefficient (R_ICC_) and 95% CI. The interpretation of R_ICC_ was as follows; poor (< 0.50), moderate (0.50–0.75), good (0.76–0.90), and excellent (> 0.90) reliability.

The statistical analyses were performed using the SPSS 19.0 package (SPSS Inc., Chicago, Illinois) and MedCalc version 15.8 (MedCalc, Ostend, Belgium). A P-value < 0.05 was regarded as statistically significant.

## Results

### Demographic characteristics of study population

The demographic information and clinical risk factors of a total of 144 patients are summarized in Table 1. The mean age of the patients was 57.7±16.5 years, and 63 patients (42.6%) were male. The prevalence of diabetes mellitus, hypertension, dyslipidemia, and current smoking was 29 patients (19.6%), 61 patients (41.2%), 33 patients (23.3%), and 11 patients (7.4%), respectively. Among them, 22 patients (14.9%) had pulmonary thromboembolism (PTE), while 18 patients (12.2%) had coronary artery disease (CAD). Based on an mPAP of 20 mmHg by RHC, 116 patients (80.2%) were diagnosed with PH, while 28 patients (19.4%) were normal.

**Table 1:** Patient characteristics.

Patients’ characteristics	n = 144
Age (years)	57.7±16.5
Gender, male	63 (42.6%)
BMI (Kg/m^2^)	24.2±4.7
Systolic blood pressure (mmHg)	121.9±18.1
Diastolic blood pressure (mmHg)	72.7±11.5
Diabetes	29 (19.6%)
Hypertension	61 (41.2%)
Dyslipidemia	33 (22.3%)
Smoking	
• current smoker	11 (7.4%)
• ex-smoker	37 (25.7%)
Small airway disease	17 (11.5%)
Pulmonary thromboembolism	22 (14.9%)
Coronary artery disease	18 (12.2%)
Right heart catheterization	
mPAP ≤ 20 mmHg	28 (19.4%)
mPAP > 20 mmHg	116 (80.2%)

Note–BMI, basal metabolic index; COPD, chronic obstructive pulmonary disease; LV, left ventricle.

### Changes in CT image parameters based on mPAP value

Table 2 summarizes the correlation of CT imaging parameters with mPAP. In large vessel measurements, all pulmonary artery diameters, including the main pulmonary artery, RPA and LPA, and the P/A ratios increased statistically significantly with increasing mPAP (all, p < 0.05). However, the diameters of the ascending aorta and IVC, both long and short, did not correlate with mPAP values.

**Table 2:** CT imaging parameters correlated with mean pulmonary artery pressure.

	Correlation coefficient (r)	P-value
***Large vessel measurement***		
Ascending aorta (mm)	-.098	0.245
Main pulmonary artery (mm)	0.416	<0.001**
P/A ratio	0.442	<0.001**
RPA (mm)	0.266	0.001**
LPA (mm)	0.274	<0.001**
IVC (long, mm)	0.156	0.063
IVC (short, mm)	0.122	0.148
***Cardiac chamber measurement***		
RV (mm)	0.445	<0.001**
LV (mm)	-0.191	0.022*
RV/LV ratio	0.502	<0.001**
RA (mm)	0.268	0.001**
LA (mm)	-0.054	0.524
RA/LA ratio	0.271	0.001**
Septal angle (degree)	0.222	0.008**

Note- RPA, right pulmonary artery; LPA, left pulmonary artery; IVC, inferior vena cava; RV, right ventricle; LV, left ventricle; RA, right atrium; LA, left atrium

** p-value < 0.01

* p-value <0.5

In the analysis of chamber dimensions, the RV diameter, RA diameter, RV/LV ratio, RA/LA ratio, and septal angle increased proportionally with increasing mPAP values (all, p < 0.05). However, the LV diameter showed a statistically inverse relationship with mPAP values, and LA diameters had no significant relationship with mPAP values. S1 Fig shows various graphs illustrating the trend of each CT imaging parameter with increasing mPAP values.

### CT image biomarkers to predict MACE

During a median follow-up of 36±27 months (range; 1–115 months), a total of 44 (30.6%) MACE occurred, comprising 14 fatalities and 30 hospitalizations due to exacerbated PH.

To investigate independent predictors of MACE, we conducted univariate and multivariate Cox analyses, incorporating demographic characteristics and various CT imaging findings (Table 3). Age (HR 1.02, 95% CI;1.00–1.05), PTE (HR 2.43, 95% CI: 1.24–4.74) and RV/LV ratio (HR 2.12, 95% CI: 1.25–4.68) were all significant univariate predictors of the MACE (p<0.05). In the multivariate Cox regression analysis adjusted for clinically significant univariate predictors, the RV/LV ratio (HR 2.32; 95% CI: 1.17–4.59) and age (HR 1.03, 95% CI;1.00–1.05) were independent predictors of MACE. Notably, PTE did not retain predictive significance in the multivariate Cox regression models.

**Table 3:** Univariate and multivariate Cox regression analysis for predicting the MACE.

	Univariate analysis		Multivariate analysis	
	HR (95% CI)	p-value	HR (95% CI)	p-value
Age	1.02 (1.00–1.05)	0.024*	1.03 (1.00–1.05)	0.022*
Male gender	0.99 (0.54–1.85)	0.996		
BMI	0.95 (0.89–1.01)	0.112		
Hypertension	1.80 (0.98–3.30)	0.055		
DM	1.53 (0.75–3.13)	0.240		
Smoking	0.35 (0.05–2.53)	0.297		
Dyslipidemia	0.85 (0.41–1.78)	0.666		
Small airway disease	1.77 (0.74–4.22)	0.201		
PTE	2.43 (1.24–4.74)	0.009*	1.91 (0.95–3.83)	0.068
Coronary artery disease	0.71 (0.26–2.00)	0.520		
Ascending aorta (mm)	1.01 (0.95–1.06)	0.802		
Main PA (mm)	0.98 (0.94–1.03)	0.487		
P/A ratio	0.86 (0.26–2.87)	0.809		
RPA (mm)	0.99 (0.94–1.05)	0.797		
LPA (mm)	0.99 (0.93–1.07)	0.927		
IVC	0.96 (0.90–1.01)	0.099		
RV (mm)	1.02 (0.99–1.05)	0.195		
LV (mm)	0.98 (0.95–1.01)	0.116		
RV/LV ratio	2.12 (1.25–4.68)	0.009*	2.32 (1.17–4.59)	0.016*
RA (mm)	1.01 (0.98–1.03)	0.620		
LA (mm)	1.01 (0.99–1.03)	0.463		
RA/LA ratio	0.99 (0.94–1.03)	0.527		
septal angle (degree)	1.00 (0.98–1.03)	0.822		

Note- BMI, basal metabolic index; DM, diabetes mellitus; PTE, pulmonary thromboembolism; PA, pulmonary artery; P/A ratio, pulmonary artery to ascending aorta diameter ratio; RPA, right pulmonary artery; LPA, left pulmonary artery; IVC, inferior vena cava; RV, right ventricle; LV, left ventricle; RA, right atrium; LA, left atrium.

**Fig 3:**
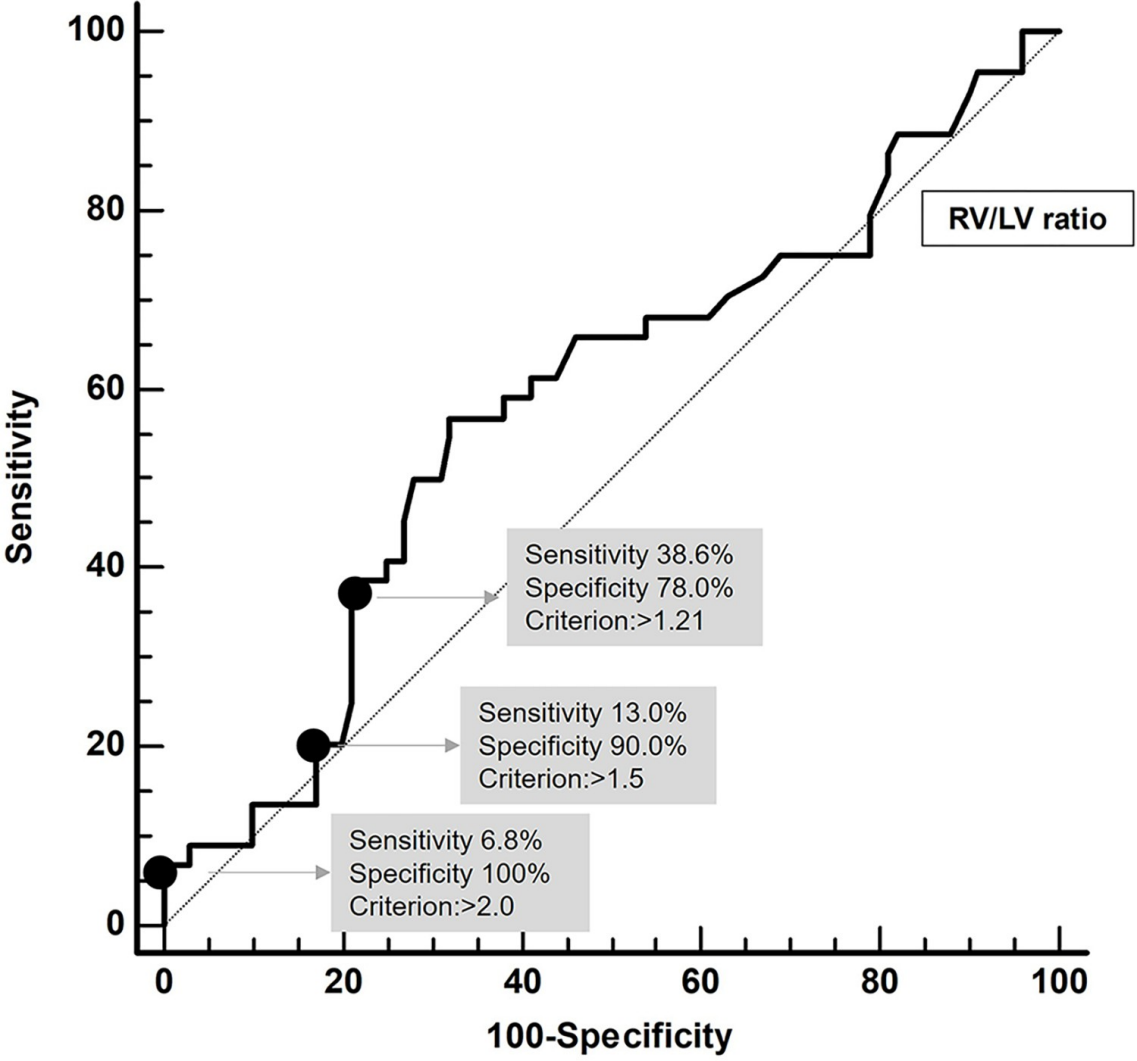
The ROC curve to determine the optimal cut-off values when predicting MACE.

In the ROC curve analysis for predicting MACE based on the RV/LV ratio, the specificity was 78% when the cut-off value of RV/LV ratio was 1.2. Subsequently, the specificity increased to 90% and 100.0% when the cut-off values for the RV/LV ratio were 1.5 and 2.0, respectively (Fig 3).

### Interobserver agreement for CT imaging parameters

The inter-observer agreement of CT imaging parameters is summarized in Table 4. Analysis of measurements for the diameters of large vessels revealed excellent inter-observer agreement for the ascending aorta (R_ICC_ = 0.96), main PA (R_ICC_ = 0.95), P/A ratio (R_ICC_ = 0.95), and RPA (R_ICC_ = 0.96). However, the inter-observer agreement for each chamber size, right to left chamber ratio and septal angle ranged from good to moderate (R_ICC_ = 0.75–0.89), which were lower compared to those of large vessels such as the main pulmonary artery and aorta.

**Table 4:** Inter-observer agreement of CT imaging parameters.

	R_ICC_	95% CI
Ascending aorta diameter	0.96	0.95–0.97
Main PA diameter	0.95	0.93–0.96
P/A ratio	0.95	0.93–0.97
RPA diameter	0.96	0.95–0.97
LPA diameter	0.86	0.81–0.90
IVC (long dimension)	0.79	0.73–0.85
IVC (short dimension)	0.81	0.74–0.86
RV diameter	0.80	0.73–0.85
LV diameter	0.89	0.86–0.92
RV/LV ratio	0.86	0.81–0.90
RA diameter	0.81	0.75–0.86
LA diameter	0.89	0.86–0.92
RA/LA ratio	0.75	0.69–0.83
Septal angle	0.84	0.78–0.88

Note- R_ICC_, intraclass correlation coefficient; CI, confidence interval; PA, pulmonary artery; P/A ratio, pulmonary artery to ascending aorta diameter ratio; RPA, right pulmonary artery; LPA, left pulmonary artery; IVC, inferior vena cava; RV, right ventricle; LV, left ventricle; RA, right atrium; LA, left atrium

## Discussion

In this study aimed at identifying effective CT imaging biomarkers for predicting risk in pulmonary hypertension patients, the key findings were as follows: 1) main PA diameter, P/A ratio, RV and RA diameter, RV/LV ratio, and RA/LA ratio increased linearly with rising mPAP values, 2) in multivariate Cox regression analysis, RV/LV ratio (HR 2.32; 95% CI: 1.17–4.59) emerged as the best predictor of MACE, followed by age (HR 1.03, 95% CI;1.00–1.05), and 3) among various CT imaging parameters, large vessel sizes including diameter of A, P, and P/A ratio exhibited excellent inter-observer agreement, while chamber size showed good to fair reliability.

Numerous studies have investigated CT imaging for diagnosing PH, particularly focusing on main PA diameter and the P/A ratio, which have shown efficacy as CT parameters [7–10, 15, 16]. Prior research using the P/A ratio to diagnose PH demonstrated a specificity of 92% for a P/A ratio > 1 [17]. Our study also found that the P/A ratio not only reflected PH diagnosis but also correlated with the severity of mPAP values, suggesting its reliability as a marker for PH severity. While previous studies have explored CT imaging parameters for the diagnosis of PH, few have addressed PH severity. While previous studies have explored CT imaging parameters for pulmonary hypertension diagnosis, few have addressed pulmonary hypertension severity. Thus, our study’s advantage lies in identifying a non-invasive CT imaging parameter reflecting the degree of mPAP measured by invasive right heart catheterization (RHC). Our findings suggest that the main PA diameter and P/A ratio exhibit excellent inter-observer reliability and can serve as efficient CT imaging markers for predicting PH severity.

The RV/LV ratio has emerged as a significant predictor of PH in previous studies [11, 16]. Meinel et al. [18] reported that the RV/LV diameter ratio measured on transverse sections was associated with an approximately 2.5-fold risk for all-cause mortality in meta-analysis of 49 studies involving 13,162 patients with acute pulmonary embolism. In our study, the RV/LV ratio not only well reflected PH severity but also emerged as the strongest predictor of MACE.

The inter-observer agreement for each chamber size was lower compared to that of large vessels such as main pulmonary artery and aorta. This difference may be attributed to the fact that CTPA is a non-ECG gated technique, resulting in greater changes in chamber dimensions compared to the movement of large vessels during the cardiac cycle. Therefore, using an ECG-gated technique could enhance the reliability of chamber dimension measurements. Additionally, our study found that the specificity for MACE was 78% at an RV/LV ratio cutoff of 1.2 and increased to 100% at a cutoff of 2. This suggests that a higher RV/LV ratio may indicate worsening right ventricular dysfunction, which could lead to severe complications or an elevated risk of mortality. Therefore, if the RV/LV ratio is elevated, it is advisable to consider prompt and proactive treatment.

Recently, some studies have suggested that the diagnostic accuracy of PH improves when various CT imaging parameters are combined [9, 12]. Swift et al. [12] demonstrated that CT diagnostic models integrating multiple metrics (such as PA diameter, interventricular septal angle, RV outflow tract thickness, and LV area) outperform individual metrics in predicting PH likelihood. Although our study did not assess combinations of different variables, our goal was to identify efficient CT parameters as simple and reproducible imaging biomarkers for predicting PH severity. The assessment of PH severity necessitates a thorough evaluation using a combination of clinical data, including biochemical markers, exercise testing, imaging (such as echocardiogram or cardiac MRI), and hemodynamic assessment. However, PH may not be accurately diagnosed in situations where a multidisciplinary team approach is unavailable or in patients with non-specific symptoms. Therefore, identifying a non-invasive and simple CT imaging parameter that reflects PH severity could help identify clinically overlooked PH patients and provide guidance for further evaluation.

This study has several limitations. Firstly, it had a relatively small sample size of 144 participants and was conducted at a single center. Although the results of this study were consistent with previous research, there is a need for future studies with larger sample sizes to improve reliability. Secondly, the enrolled PH patients underwent both RHC and CTPA, resulting in a heterogenous group with diverse PH causes. To reduce heterogeneity, patients with congenital heart disease with large shunt, severe valvular disease or LV dysfunction were excluded, but further specific research focusing on particular PH causes is necessary. Thirdly, since CTPA lacks ECG gating, chamber size measurements may vary. However, the inter-observer agreement for the RV/LV ratio, which assesses relative changes between chambers, was good. Future research should examine how chamber sizes in non-ECG gated CTPA compare to those in ECG-gated coronary CT angiography to assess the extent of differences.

In conclusion, among CT parameters, the RV/LV ratio was the strongest predictor of MACE in patients with PH, while the P and P/A ratios were the reliable indicators reflecting mPAP levels.

## Supporting information

S1 Fig Various graphs that showing the trend of each CT imaging parameter according to mPAP values. (**A**) Main PA diameter, (**B)** Ascending aorta diameter, (**C)** P/A ratio, (**D)** IVC long dimension (**E**) RA dimension, (**F)** LA dimension, (**G)** RA/LA ratio, (**H)** RV dimension, (**I)** LV dimension, (**J)** RV/LV ratio, (**K**) Septal angle.
(TIF)
