# Supplementary figures and images for: CT imaging biomarkers to predict severity and prognosis of pulmonary hypertension
Source: PLoS One. 2025 Feb 12;20(2):e0313235. doi: 10.1371/journal.pone.0313235 (PMC11819466; doi:10.1371/journal.pone.0313235)

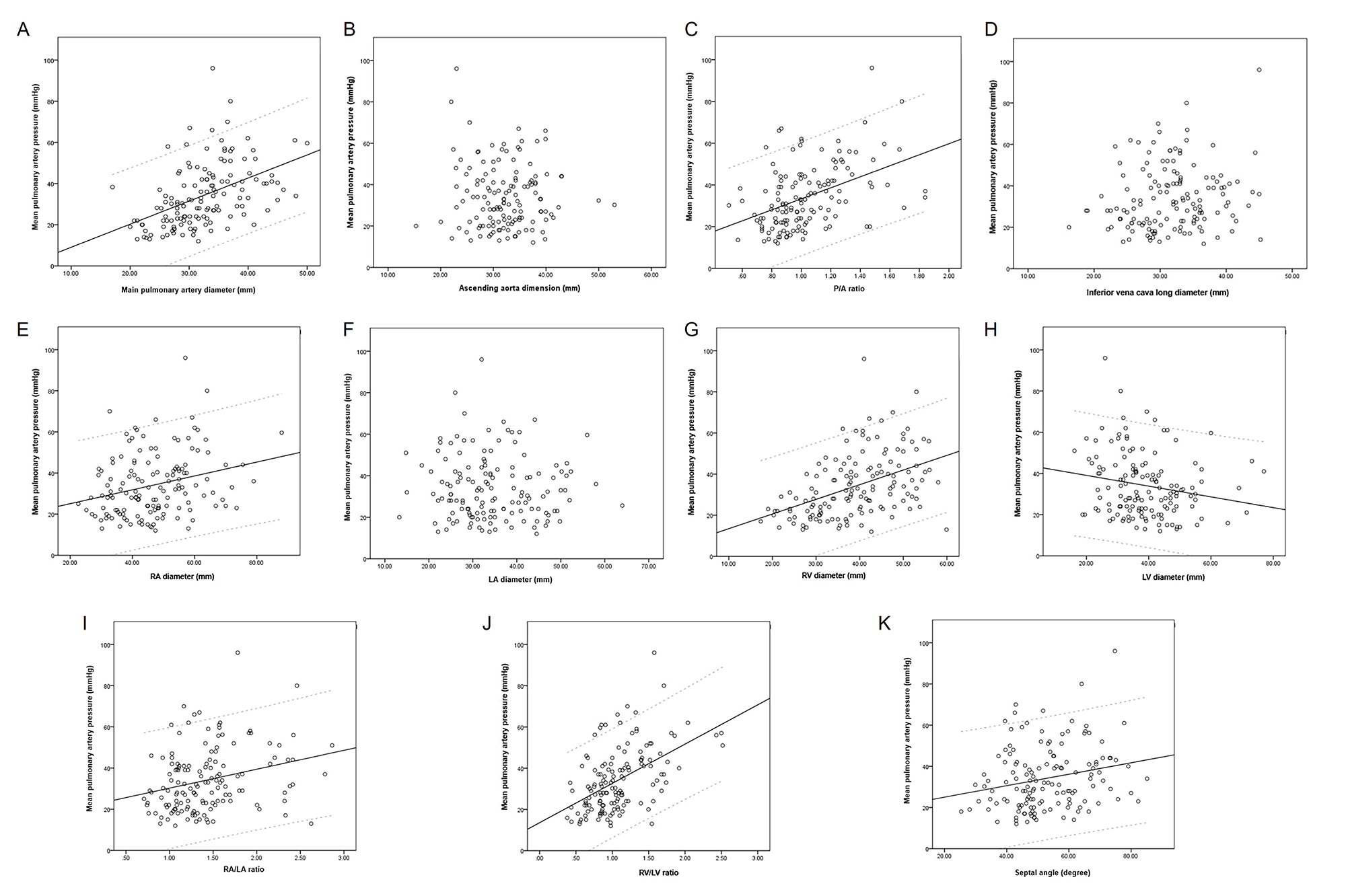

Supplement: S1 Fig — (A) Main PA diameter, (B) Ascending aorta diameter, (C) P/A ratio, (D) IVC long dimension (E) RA dimension, (F) LA dimension, (G) RA/LA ratio, (H) RV dimension, (I) LV dimension, (J) RV/LV ratio, (K) Septal angle. (TIF) [file pone.0313235.s001.tif]
